# Retraction: Molecular characterization of leaf spot caused by *Alternaria alternata* on buttonwood (*Conocarpus erectus* L.) and determination of pathogenicity by a novel disease rating scale

**DOI:** 10.1371/journal.pone.0272185

**Published:** 2022-08-03

**Authors:** The PLOS ONE Editors

The *PLOS ONE* Editors retract this article [1] because it was identified as one of a series of submissions for which we have concerns about authorship, competing interests, and peer review. We regret that the issues were not addressed prior to the article’s publication.

AMAS agreed with the retraction. MR, SA, SAA and MA either did not respond directly or could not be reached. MFA and MJA did not agree with the retraction.
